# ENERGY INTAKE FROM ULTRA-PROCESSED FOODS AMONG ADOLESCENTS

**DOI:** 10.1590/1984-0462/;2017;35;1;00001

**Published:** 2017

**Authors:** Helen Freitas D’Avila, Vanessa Ramos Kirsten

**Affiliations:** a Universidade Federal de Santa Maria (UFSM), Palmeira das Missões, RS, Brasil.

**Keywords:** Adolescent, Industrialized food, Nutritional status

## Abstract

**Objective:**

To evaluate the consumption of ultra-processed foods and related factors in adolescents.

**Methods:**

This is a cross-sectional study conducted with 784 adolescents (both sexes and aged between 12 and 19 years) from public and private schools in the municipality of Palmeira das Missões, Brazil. Food consumption was recorded by the semiquantitative questionnaire of frequency of food consumption and converted to energy (kcal/day). Foods were classified as minimally processed, group 1 (G1); processed foods, group 2 (G2); and ultra-processed foods, group 3 (G3). The variables evaluated were sex, socioeconomic class, color, physical activity, body mass index, and blood pressure levels. In the comparison of quantitative variables, the Mann-Whitney test and the Kruskal-Wallis H test were used. To adjust the differences between the groups, considering the effects of total calories, the covariance analysis test (ANCOVA) was applied.

**Results:**

The median of the total energy consumption was 3,039.8 kcal, and that of ultra-processed foods was 1,496.5 kcal/day (49.23%). The caloric intake from foods in G1, G2, and G3 did not differ according to the skin color of the adolescents. Those belonging to socioeconomic classes C and D are the most frequent consumers of calories from G2 and G3 (p<0.001). Underactive teens consume fewer calories from minimally processed foods. Eutrophic adolescents present higher consumption of G3 foods (p<0.001) when compared to those who are overweight.

**Conclusions:**

The consumption of ultra-processed foods was associated with socioeconomic level, physical activity level, and nutritional status.

## RESUMO

**Objetivo:**

Avaliar o consumo proveniente dos alimentos ultraprocessados e fatores relacionados em adolescentes.

**Métodos:**

Estudo transversal com 784 adolescentes (de ambos os sexos e entre 12 a 19 anos) de escolas públicas e privadas do município de Palmeira das Missões, Brasil. O consumo alimentar foi registrado pelo questionário semiquantitativo de frequência de consumo de alimentos e convertido em energia (Kcal/dia). Os alimentos foram classificados em: minimamente processados, grupo 1 (G1); alimentos processados, grupo 2 (G2); e alimentos ultraprocessados, grupo 3 (G3). As variáveis avaliadas foram: sexo, classe socioeconômica, cor, atividade física, índice de massa corpórea (IMC) e níveis pressóricos. Na comparação de variáveis quantitativas foi usado o teste de Mann-Whitney e o teste H de Kruskal-Wallis. Para ajustar as diferenças entre os grupos, considerando os efeitos de calorias totais, foi aplicado o teste de análise de covariância (ANCOVA).

**Resultados:**

A mediana do consumo energético total foi de 3.039,8 Kcal e a de ultraprocessados foi de 1.496,5 Kcal/dia (49,23%). O consumo calórico proveniente dos alimentos do G1, do G2 e do G3 não diferiu de acordo com a cor da pele dos adolescentes. Os adolescentes pertencentes às classes C e D são os maiores consumidores de calorias do G2 e do G3 (p<0,001). Os adolescentes insuficientemente ativos consomem menos calorias de alimentos minimamente processados. Os adolescentes eutróficos apresentam maior consumo do G3 (p<0,001), quando comparados aos que possuem excesso de peso.

**Conclusões:**

O consumo de alimentos ultraprocessados associou-se ao nível social, ao nível de atividade física e ao estado nutricional.

## INTRODUCTION

In Latin America, dietary patterns have undergone profound changes in recent years. In Brazil, for example, between 2002-2003 and 2008-2009, there was an increase in the availability of ready-to-eat products (23-27.8% of calories), as a result of the increase in consumption of ultra-processed products (from 20.8% to 25.4%) in all income classes. During this period, there was a significant decrease in consumption of minimally processed foods and cooking ingredients.^1^

The feeling of not having enough time is related to changes in food consumption patterns, such as reduced time spent preparing food at home, increased consumption of ready-to-eat foods, and decreased food consumption in the whole family.^2^ ^,^ ^3^ This type of foods, often cheaper than fresh foods, offer highly energetic and palatable meals^1^ and are associated with less healthy diets, contributing to the emergence of obesity and chronic health problems.^3^

In order to describe dietary patterns and to identify how they may affect health, Monteiro et al.^4^ propose a new classification covering three groups according to the extent and purpose of the treatment used in their production: group 1 consists of unprocessed and minimally processed foods; group 2, for processed foods or foods that serve as culinary ingredients; group 3 involves ultra-processed, ready-to-eat or frozen food with little or no preparation. It is important to emphasize that a diet made only with products of the third group is twice as dense in energy when compared to the diet prepared with food from the other groups.^5^

The change in the dietary profile of the population did not occur only in adults.^6^ However, few studies have shown the relationship of excess weight and metabolic changes^5^ with high ingestion of ultra-processed foods.^4^ ^,^ ^7^ These foods are generally more sugary, salty, fatty, with a high glycemic load, and dense energy when compared to freshly prepared foods, meals, and dishes made with unprocessed or minimally processed foods and cooking ingredients.^5^ ^,^ ^8^

Thus, the objectives of this study were to evaluate the caloric intake from ultra-processed foods and verify their association with socioeconomic factors, nutritional status, level of physical activity, and blood pressure levels.

## METHODS

This is a cross-sectional study carried out from September 2013 to October 2014 in Palmeira das Missões (RS), a country town located in the northwest of the state of Rio Grande do Sul, with residents belonging to various ethnic groups and a current population of 34,328 inhabitants. The sector that drives the local economy is agriculture and livestock. Palmeira das Missões presents a human development index (HDI) of 0.737, according to data from the Brazilian Institute of Geography and Statistics (IBGE) of 2010.^9^

The sample was considered as non-probabilistic (convenience), with the participation of 784 adolescents, out of a total of 1,317 enrolled (representing approximately 60% of the studied population), from all schools (public and private) located in the central region of the municipality. By performing a sample calculation *a posteriori*, the sample size recommendation of 710 students was obtained, taking into account the population of 1,317 students enrolled, an estimated sample error of 2.5%, a 95% confidence level, and a more homogeneous profile of population distribution. The inclusion criterion of the schools was the presence of 8th grade and high school classes. As a criterion for the inclusion of adolescents, those who were in 8th grade of elementary school and the 3rd year of high school were considered, including students and parents/guardians who expressed their consent to the participation of adolescents in the study. Students who were not present on the days of data collection and those who refused to be part of the investigation were considered as losses. Pregnant women, students with dwarfism, exchange students, students with bone fractures, and those with hearing problems were excluded.

To determine the regular food consumption of adolescents, a semiquantitative food consumption frequency questionnaire (QFCA) was elaborated for adolescents from the metropolitan region of Rio de Janeiro, with 90 items.^10^ With the application of the pilot test, we noticed the need to remove some non-traditional foods from this population, such as croissant, and to change the terminology of some foods, such as hamburgers (not traditional in the state of Rio Grande do Sul). Also, the age group that comprised the QFCA was more clearly delineated, excluding students from school years below the 8th grade. The frequency records of food consumption in the QFCA were converted to energy (kcal/day) consumed, using the Brazilian Food Composition Table.^11^ For the foods that were not found in the mentioned table, the Nutritional Composition Table of Foods Consumed in Brazil was used.^12^ Also, for foods not found (cheese salad burgers, instant soup, industrialized juice box/package/bottle) in none of the tables cited, nutritional information were taken from labels searched on the internet.

For data analysis, 174 students were excluded, for whom the energy consumption was higher than 6,000 kcal or less than 500 kcal.^13^ The variables evaluated were sex, socioeconomic class, self-reported skin color, physical activity level, body mass index (BMI), and blood pressure levels.

Foods were classified into three groups, according to the level of processing.^14^ Group 1 (G1) was represented by unprocessed or minimally processed foods, such as fresh meat, milk, cereals, fruits, and vegetables. Group 2 (G2) was composed of processed foods used as ingredients of culinary preparations, such as oils and fats, flours, pasta, starch, and sugars. Group 3 (G3) consists of ultra-processed foods, such as breads, biscuits, ice creams, chocolates, candies/sweets, snacks, potato chips, sweetened drinks such as sodas, as well as meat products such as nuggets, hot dogs, burgers, and luncheon meats.

Self-reported information on sex and skin color (white, brown, and black) was collected by means of a questionnaire. Students who declared themselves to be Asian (0.4%) and indigenous (1.6%) were excluded from the statistical analysis because of their low percentage. For statistical purposes, blacks and browns were analyzed together in the elaborated comparisons.

The socioeconomic classification followed the criteria of the Brazilian Association of Research Companies (Abep) and was presented in classes, named A1, A2, B1, B2, C1, C2, D, and E, corresponding respectively to a given score.^15^ For data analysis, classes A1 and A2, B1 and B2, C1 and C2, and D were grouped together.

The level of physical activity was evaluated using the criteria and grouping in categories proposed by the International Physical Activity Questionnaire (Ipaq). This questionnaire was reproduced and validated for Brazilian adolescents^16^ and classifies the sample into three categories: insufficiently active, sufficiently active, and very active.

To evaluate the nutritional status by means of BMI, the World Health Organization’s recommendation was followed,^17^ and all anthropometric measurements were performed in duplicate. Data collected were birth date, weight (kg), and height (cm). The BMI was calculated using the WHO AnthroPlus software (version 3.2.2)^18^ and the nutritional status classification was calculated according to the BMI percentiles for age and sex.^19^ Adolescents were considered overweight/obese when the percentile was greater than or equal to 85, and non-obese when the percentile was below 85. A prevalence of 2.9% of adolescents with low weight was found. For statistical purposes, such individuals were grouped with eutrophic adolescents.

Pressure levels were measured using duly calibrated automatic equipment Omron 705 CP-II, which proved to be valid for the measurement of young individuals, according to a study performed with a Brazilian population.^12^ Two consecutive readings were recorded at 2-minute intervals, using the second for classification. This followed values referring to the 90th, 95th, and 99th percentile of blood pressure levels for adolescents, according to age, sex, and percentiles of height of those evaluated. Values below the 90th percentile were considered normal; values between the 90th and 95th percentile, borderline; values equal to or above the 95th percentile were defined as increased.

This study was approved by the Research Ethics Committee of *Universidade Federal de Santa Maria* (UFSM) under protocol number 19984713.1.0000.

The data were analyzed using statistical software Statistical Package for the Social Sciences (SPSS) version 18.0. Quantitative data were described by mean ± standard deviation and categorical data, by percentage. In the presence of asymmetry, median and interquartile range were used. The asymmetry of the variables was tested using the Kolmogorov-Smirnov test. In the comparison of quantitative variables, the Mann-Whitney test and Kruskal-Wallis *H* test were used. To adjust the differences between the groups, taking into account the effects of total calories, the covariance analysis test (ANCOVA) was used. Statistically significant values were considered when *p*<0.05.

## RESULTS

The sample consisted of 784 adolescents with a mean age of 15.25±1.26 years. The female sex constituted 57.4% (n=450) of the sample. Students belonging to the socioeconomic class B1 and B2 represented 60.1% (n=463) of the sample, and the white color was self-declared by 62.4% (n=471) of participants. Most of the sample was considered very physically active (57.4%, n=450), with normal pressure levels (75%, n=560), and eutrophic (76.6%, n=573). The median of total energy consumption was 3,039.8 kcal and that of ultra-processed foods was 1,496.5 kcal/day, representing 49.2% (Table 1).

**Table 1:**
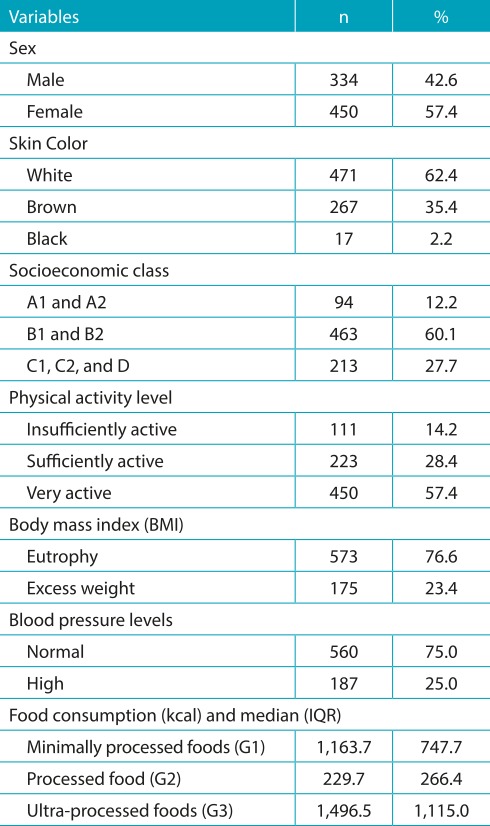
Sociodemographic characteristics, level of physical activity, body mass index, blood pressure levels, and food consumption of adolescents from Palmeira das Missões, RS, Brazil (2013-2014).

IQR: interquartile range.

Adolescents who declared themselves to be brown or black (analyzed together) consumed more calories from G1 (*p*=0.021) and G3 (*p*=0.045), when compared to white adolescents. However, after adjusting for total calories, the difference lost significance (data not shown). Adolescents belonging to classes C and D consumed more products from G2 (*p*<0.001) and G3 (*p*<0.001), compared to those belonging to classes A and B (Table 2). In the comparison of the energy consumption coming from the groups (G1, G2, and G3) according to the sex of the adolescents, there was no statistical difference (data not shown).

**Table 2:**
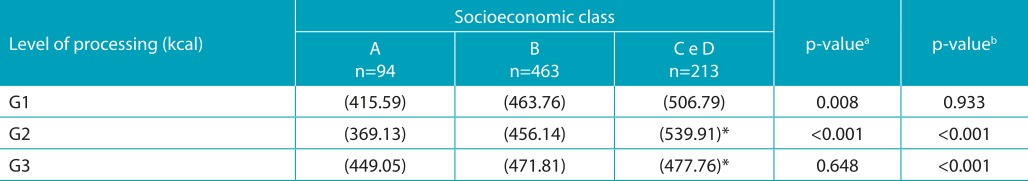
Energy consumption of minimally processed foods (G1), processed foods (G2), and ultra-processed foods (G3) according to the socioeconomic classification of adolescents from Palmeira das Missões, RS, Brazil, from 2013 to 2014.

^a^Kruskal-Wallis H test; ^b^ *p*-value for ANCOVA. Means adjusted for total caloric value.

Table 3 shows that insufficiently active adolescents consumed less energy from minimally processed foods compared to those who were sufficiently active and very active. No difference was observed in the other groups (G2 and G3) with respect to physical activity. It was also verified that eutrophic adolescents presented higher consumption of ultra-processed foods (*p*<0.001), when compared to adolescents who were overweight (Table 4). There was no association of food consumption in view of its degree of processing with blood pressure levels (data not shown).

**Table 3:**
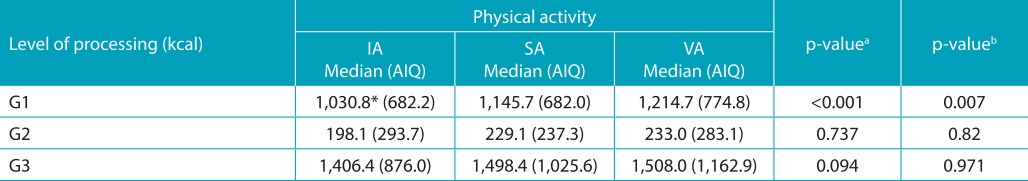
Energy consumption of minimally processed foods (G1), processed foods (G2), and ultra-processed foods (G3) according to the level of physical activity of adolescents, Palmeira das Missões, RS, Brazil, 2013-2014.

IA: insufficiently active; SA: sufficiently active; VA: very active; IQR: interquartile range; ^a^Kruskal-Wallis H test; ^b^ *p*-value for ANCOVA. Means adjusted for total caloric value.

**Table 4:**
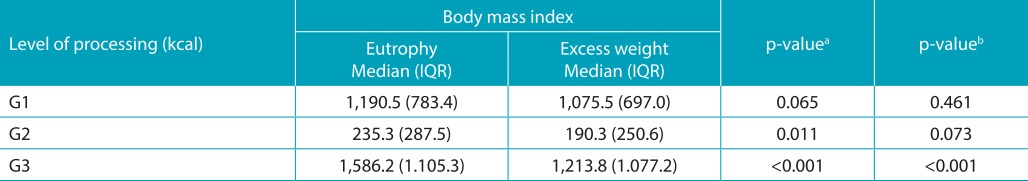
Kcal intake from minimally processed foods (G1), processed foods (G2), and ultra-processed foods (G3) according to the body mass index of adolescents, Palmeira das Missões, RS, Brazil, 2013-2014.

IQR: interquartile range; ^a^Kruskal-Wallis H test; ^b^ *p*-value for ANCOVA. Means adjusted for total caloric value.

## DISCUSSION

This study found that adolescents, even from small, countryside towns, present high energy consumption from ultra-processed foods, and this consumption was associated with socioeconomic class, physical activity level, and nutritional status.

Food processing is a much debated issue today. Most studies^20^ ^,^ ^21^ ^,^ ^22^ ^,^ ^23^ ^,^ ^24^ use the terminology “fast food” for industrialized foods, but Monteiro et al.^4^ created a new classification of foods into three groups, in line with the level of processing, with the third group comprising foods that have been processed, those that require little or no preparation. The new Food Guide for the Brazilian population^25^ proposes a classification similar to that of Monteiro et al.,^4^ with the same terminology, differing only on breads and baked foods. Monteiro et al.^4^ consider breads as ultra-processed when, in addition to wheat flour, yeast, water, and salt, ingredients include substances such as hydrogenated vegetable fat, sugar, starch, whey, emulsifiers, and other additives.

The energy consumption from ultra-processed foods in this study was extremely high, as the food guide^25^ estimates that an average of 2,000 calories will be enough to younger people. However, Andrade et al.,^10^ when analyzed overweight and non-overweight adolescents aged between 12 and 17.9 years, also reported high intakes of foods with high energy density in adolescents in Rio de Janeiro. On the other hand, the study by Canella et al.^26^ verified low family consumption of these foods when using the analysis of food availability as a research tool. An investigation carried out with children assisted at the Basic Health Unit of Porto Alegre found a value similar to this study, with 47% of the calories coming from ultra-processed foods.^27^ The first recently published study with adults and adolescents^28^ found that the sample consumed approximately 30% of the calories from ultra-processed foods; however, this evaluation was not conducted separately, according to the age group. Evaluating the dietary intake of the Brazilian diet using the Family Budgets Survey, a slightly lower calorie value was found from ultra-processed foods (21.5%).^29^

Adolescents belonging to socioeconomic classes C and D in this study were the largest consumers of calories from processed (G2) and ultra-processed (G3) foods. Corroborating with the present study, in which the poorest participants consume more ultra-processed calories, Miqueleiz et al.^30^ studied the association between socioeconomic class and consumption in Spanish families and obtained an inverse gradient: children and adolescents from high and low socioeconomic strata, respectively, presented the lowest and highest percentage of unhealthy consumption (fast food, sugary drinks, snacks, chips, biscuits). But the results of Bielemann et al.^31^ are opposed to this study, as adults who declared themselves to have never been poor presented higher consumption of ultra-processed foods.

Fruits and vegetables found in G1 have less energy and are high in fiber, essential nutrients, and minerals. Therefore, their consumption can benefit health by reducing the total energy content of the diet, improving its nutrient density.^32^

Galvez et al.^33^ found that children living in a small area with one or more convenience stores were more likely to have a higher BMI. The adolescents in this study reside in a countryside town of Southern Brazil without fast-food chains. Limited access to this type of food, and the fact that the sample presents a high prevalence of physical activity, is perhaps the explanation for the inverse association between ultra-processed calories and BMI.

Some studies have shown an association of consumption of ultra-processed foods with overweight and obesity in adults,^26^ with metabolic syndrome in adolescents,^34^ and general and central obesity in adult women.^35^ Furthermore, it was found that in adult individuals in Guatemala, the 10% increase in the share of highly processed foods increases BMI by 4.25%, as well as the probability of excess weight and obesity.^36^ In students aged 12-20 years, strong positive associations were found between weight change and consumption of starches, refined grains, and processed foods.^37^ It is worth noting that, of the studies cited, many analyzed only some of the foods included in the ultra-processed classification.

However, when the same situation is observed in adolescents, the association previously described has not been evidenced, as reported in this study. According to a research involving Iranian adolescents, there was no significant association between junk food (fast foods and snacks) with obesity and hypertension.^38^ In addition, a cohort study conducted in Pelotas (RS) with adults showed that obese individuals ingest less calories from ultra-processed foods.^31^

This research may have found no association between the consumption of ultra-processed foods and overweight/obesity in adolescence, but it is possible that such outcomes appear in adulthood, as childhood nutritional events can determine changes in anabolic/catabolic systems and induce metabolic outcomes at more advanced ages.^39^ Moreover, the estimate of daily energy intake is not well judged when based on food surveys.^37^ In addition, because it is a cross-sectional study, there is a temporal limitation, as obesity itself can change the eating habits of adolescents.

The possibility of a calibration bias of QFCA is also necessary to highlight. QFCA may be less accurate for adolescents, as this age group sometimes have difficulties in estimating portion sizes and food intake.^40^ Although it is feasible, the QFCA makes no distinction between food groups G1, G2, and G3, and the reports of intake in times per day, week, or month were converted into calories per day for consumption analyzes. Therefore, a cohort study is recommended in order to improve the understanding of the impact of the consumption of ultra-processed foods in adolescents.

It can be concluded that adolescents, even in small towns and in the countryside, present high energy consumption from ultra-processed foods and that this consumption is related to socioeconomic class, level of physical activity, and nutritional status.

Ultra-processed foods have abundant television advertising in programs aimed at reaching adolescents; however, these programs rarely show the consequence of the high consumption of this type of food. Therefore, it would be prudent for the government to adopt relevant legislations and regulations on advertisements and processed foods to change this scenario.
